# Approach Study for Mass Balance of Pesticide Residues in Distillers’ Stillage along with Distillate and Absence Verification of Pesticides in Distilled Spirits from Pilot-Scale of Distillation Column

**DOI:** 10.3390/molecules24142572

**Published:** 2019-07-15

**Authors:** Jung-Ah Shin, Hoonho Cho, Dong-Woo Seo, Hee-Gon Jeong, Sung Chul Kim, Jeung-Hee Lee, Soon-Taek Hong, Ki-Teak Lee

**Affiliations:** 1 Department of Food Science and Technology, Chungnam National University, 99 Daehak-ro, Yuseong-gu, Daejeon 34134, Korea; 2 Chilseo Ethanol Factory, IL SAN Trading Co., 551 Daebu-ro, Chilseo-myeon, Haman-gun, Gyeongsangnam-do 52001, Korea; 3 Department of Bio Environmental Chemistry, Chungnam National University, 99 Daehak-ro, Yuseong-gu, Daejeon 34134, Korea; 4 Department of Food and Nutrition, Daegu University, 201 Daegudae-ro, Gyeonsan-si, Gyeongsangbukdo 38453, Korea

**Keywords:** lead and cadmium, pesticides, mass balance, distillate and distillers’ stillage, distilled spirits, distillation column

## Abstract

Herein, contaminants remaining in distillate and distillers’ stillage were quantitatively measured after distillation. After rice bran powder was contaminated with 10 ppm of lead (Pb) and cadmium (Cd) or 0.02–1.27 ppm of five pesticides (terbufos, fenthion, iprobenfos, flutolanil, and ethoprophos) followed by fermentation, single-stage distillation was performed. In the obtained distillate, no Pb or Cd was found, as expected. However, when the pesticides were added as contaminants, trace–0.05 ppm of some pesticides were detected in the distillate, possibly due to the high vapor pressure (e.g., that of ethoprophos) and contamination amount (e.g., that of flutolanil, terbufos, and fenthion). In contrast, none of the contaminating pesticides were observed in the distilled spirits when a fermented liquefaction contaminated with 0.04–4 ppm of six pesticides (fenthion, terbufos, ethoprophos, iprobenfos, oxadiazon, and flutolanil) was distilled using a pilot-plant scale distillation column, indicating that the pesticides hardly migrate to the distilled spirits.

## 1. Introduction

Generally, distilled spirits are produced from grains containing carbohydrates (i.e., starch) and refer to neutral alcohol that is composed of at least 95 vol% ethyl alcohol in the EU and the United States [1,2]. In South Korea, distilled spirits (at least 95 vol% ethyl alcohol) are usually produced from rice, sweet potatoes, and tapioca as raw materials after fermentation and distillation processes. Therefore, in the case of distilled spirits, the raw materials are finally converted into ethanol, in which none of the characteristics of the raw material remains after the manufacturing process.

When cultivating agriculture crops used as raw materials of distilled spirits, pesticides are used for some purposes. If the raw materials are contaminated with pesticides, their presence in the distilled spirits would be governed by the physicochemical properties of each pesticide during the manufacturing process. Previously, Han et al. [3] reported the residue levels of five insecticides during the manufacture of sorghum distilled spirits and Cabras et al. [4] stated the residual contents of eight fungicides and five insecticides in distilled spirits of wine and associated by-products (i.e., cake and lees). In addition, Inoue et al. [5] reported the residual contents of 249 pesticides in distillates. All previous studies concluded that the possibility of contaminating pesticides remaining in distillates or distilled spirits was reduced by the actual distillation process.

Most countries oversee standards on the safe use of pesticides (e.g., the frequency of use and the allowed time of use, etc.) before harvest. These standards ensure that the amount of pesticides in agricultural products does not exceed the maximum residue limits (MRL). If the standards on safe use are disregarded and pesticides are used in excess, the MRL may be exceeded. It is critical that the pesticide amount in raw materials of all foods remains below the MRL, otherwise, the exposure to the pesticide would likely cause harm to consumers. In the case of distilled spirits, it is difficult to inspect all incoming raw materials for high pesticide levels. Therefore, distilled spirits manufacturers periodically inspect randomly selected materials. In addition, some raw materials for distilled spirits are imported from countries with low awareness of standards on safe use. While they are obeying the diversified procurement policy of raw materials, there is a possibility that manufacturers may use contaminated materials.

Some pesticides can be destroyed by biological degradation during fermentation or lost during saccharification and distillation because they can be evaporated by heat or thermally degraded [6,7,8]. Nevertheless, if a certain amount of pesticide migrates to distillates or distilled spirits, the remainder (except for the quantity lost during fermentation and distillation) must be found in the distillers’ stillage. Therefore, to obtain the mass balance, the pesticide content in the raw material, distillate, and distillers’ stillage should be quantitatively analyzed. However, this is practically difficult because homogeneous samples are difficult to obtain by mixing very small amounts of pesticides evenly within the analyte. Thus, an experimental scheme that enables obtaining a mass balance during distillation is needed to be developed.

Herein, the raw material (i.e., rice bran powder) was artificially contaminated with Pb and Cd. After fermentation, single-stage distillation was performed to obtain distillate and distillers’ stillage to determine whether all Pb and Cd remained in the distillers’ stillage. For this purpose, an experimental scheme was developed in which the entire quantities of the distillate and distiller stillage were analyzed using a minimal amount of raw material. After verifying that the proposed scheme can be used to obtain the mass balance, five pesticides were artificially added to the raw material to determine the contents of pesticides in the distillate and distillers’ stillage. Furthermore, a pilot-plant scale of the distillation column was used for distillation of the fermented liquefaction contaminated with the selected six pesticides to determine whether the pesticides migrated into the distilled spirits.

## 2. Results and Discussion

### 2.1. Determination of Pb and Cd from Single-Stage Distillation

**Figure 1:**
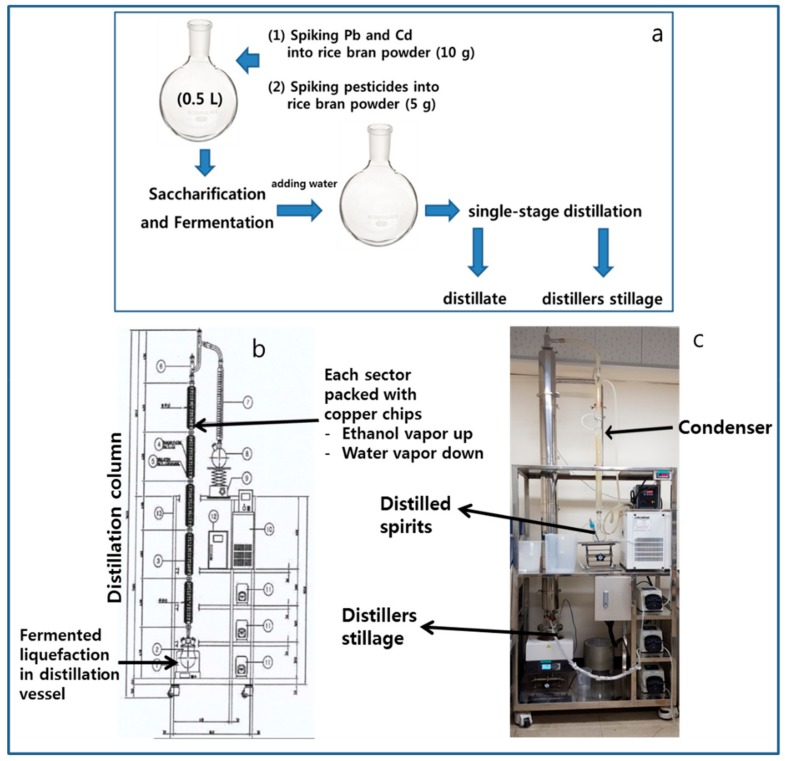
(**a**) Experimental scheme for single-stage distillation; (**b**) illustration of the pilot-plant scale distillation column; (**c**) photograph of the pilot-plant scale distillation column.

According to the experimental scheme (Figure 1), the rice bran powder (10 g) was contaminated with Pb and Cd standard solutions spiked to a final concentration of 10 ppm. Herein, 0.017 ppm Pb and 0.011 ppm Cd were detected in the raw material (i.e., rice bran powder), and the concentrations of Pb and Cd in the rice bran powder after spiking were 8.85 ppm and 9.27 ppm, respectively. However, none of the Pb and Cd was found from the distillate obtained after single-stage distillation, while Pb (10.09 ppm) and Cd (9.57 ppm) were detected in the distillers’ stillage (Table 1). According to American Organization of Analytical Chemists (AOAC), the admissible spiking recovery rates at 10 ppm are 80% to 110% [9]. When the recovery rate was defined as follows, [(content detected from distillate + content detected from distillers’ stillage)/(spiked content + content initially detected from brown rice powder) × 100], the recovery rates were 95.6–100.7%, which can be regarded as satisfactory. After analysis, the contents were expressed as distillate migration rate (DMR) and distillers’ stillage residual rate (DSRR) in Table 1. As predicted, Pb and Cd did not migrate to the distillate. The DMRs of Pb and Cd were both 0% because they were not detected in the distillate. Therefore, since the DSRR of Pb and Cd were 103–114%, most of the spiked Pb and Cd remained in the distillers’ stillage. Consequently, the scheme suggested in this experiment was used to examine the mass balance of pesticides during distillation.

### 2.2. Determination of Five Pesticides from Single-Stage Distillation

The scheme was used to investigate whether pesticides migrate during distillation. Unlike Pb and Cd, the drying step was excluded due to concerns that it would affect the mass balance. The five selected pesticides are generally used for the cultivation of rice, root, and tuber crops. The rice bran powder (5 g) was simultaneously contaminated with five pesticides by spiking and fermentation was performed thereafter (Figure 1a). The pesticide contents detected in rice bran powder after spiking, distillate, and distillers’ stillage obtained via single-stage distillation are listed in Table 2. Initially, the rice bran powder as a raw material did not contain any pesticides used in this study. In the distillate, terbufos (0.02 ppm after spiking) and iprobenfos (0.28 ppm after spiking) were not detected, while trace amounts of fenthion (0.14 ppm after spiking) and terbufos (0.04 ppm after spiking) were detected. In addition, 0.01, 0.05, and 0.01 ppm of flutolanil, ethoprophos, and fenthion (0.33 ppm after spiking) was detected, respectively (Table 2).

**Figure 2:**
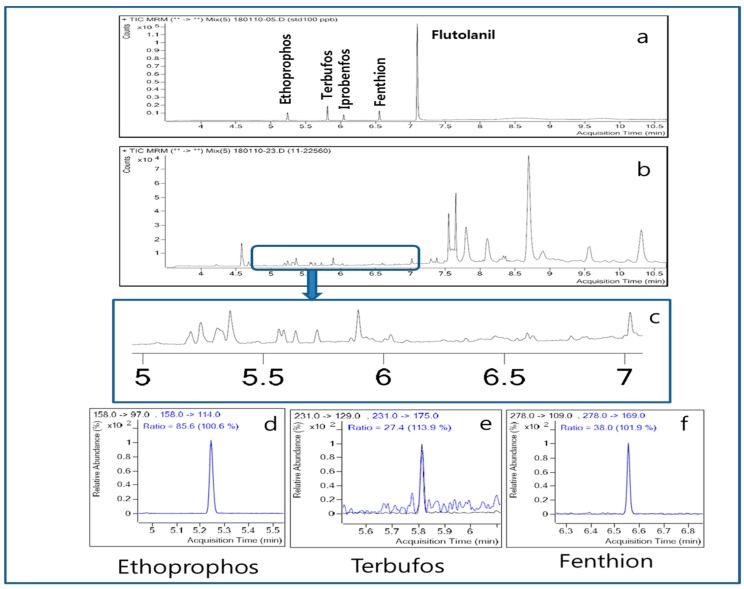
Identification of contaminating pesticides in the distillate obtained from the single-stage distillation. (**a**) Standards; (**b**) distillate; (**c**) enlarged region; (**d**) ethoprophos; (**e**) terbufos; (**f**) fenthion.

An example of the detection used herein is shown in Figure 2. The precursor ion of ethoprophos is 158 (*m/z*) and the product ions used for qualitative and quantitative analyses are 114 and 97. Since the ion pattern matching rate between the peak from the distillate and the standard was 100.6%, the peak was identified as ethoprophos (Figure 2). Therefore, it can be said that ethoprophos migrated into the distillate during the distillation. The other pesticides found in the distillate also showed sufficient ion pattern matching rates for positive identification (Figure 2). In particular, when the contamination content of terbufos was increased from 0.02 to 0.04 ppm, the amount of terbufos in the distillate increased from ND to trace (Table 2). Besides, when the contamination content of fenthion was increased from 0.14 to 0.33 ppm, the detected trace amount changed to 0.01 ppm in the distillate (Table 2). These results indicate that if the contamination content of such pesticides increases, the degree of migration into the distillate also increases. In addition, the migration degrees during distillation are dependent on the physicochemical properties of the pesticide because the DMR of flutolanil with the largest amount of contamination (i.e., 1.27 ppm) was only 0.8%, whereas that of ethoprophos with contamination of 0.35 ppm was 14.3%. Previously, Inoue et al. [5] contaminated mash with 249 pesticides including ethoprophos, fenthion, flutolanil, and iprobenfos at a concentration of 50 ng/mL, and distilled the mash under reduced pressure (100 mmHg) at 57 °C. Although most pesticides were not detected in the distillate (40% ethanol), 13.1% and 6.9% of the ethoprophos and terbufos migrated, respectively. However, if the distillation conditions are changed, different results can be obtained. When the distillation was performed at 99 °C under atmospheric pressure, 6.7–6.8% of the iprobenfos and fenthion and 15.3–28.6% of the ethoprophos and terbufos migrated into the corresponding distillates [5].

**Figure 3:**
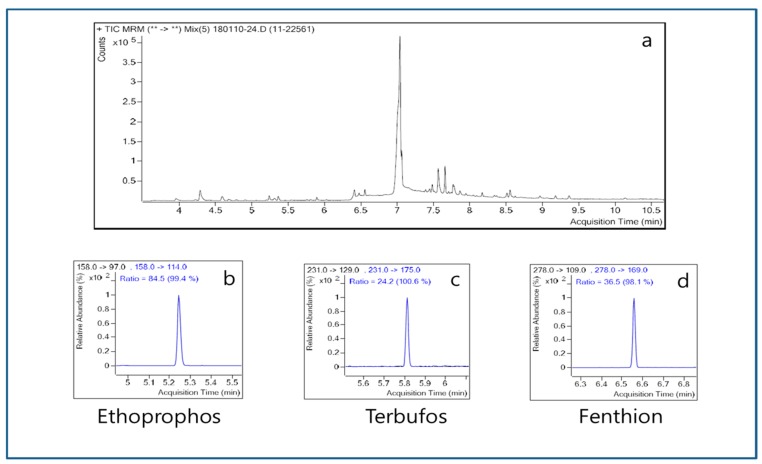
Identification of contaminating pesticides in the distillers’ stillage obtained from single-stage distillation. (**a**) Distillers’ stillage; (**b**) ethoprophos; (**c**) terbufos; (**d**) fenthion.

In the distillers’ stillage, terbufos (0.01–0.03 ppm), iprobenfos (0.4 ppm), fenthion (0.12–0.28 ppm), flutolanil (1.77 ppm), and ethoprophos (0.3 ppm) were found (Table 2). As seen in Figure 3, the ion pattern matching rates of ethoprophos, terbufos (0.04 ppm after spiking), and fenthion (0.33 ppm after spiking) were 99.4%, 100.6% and 98.1%, respectively, indicating that these pesticides remained in the distillers’ stillage. Since the DSRRs of the pesticides were 50–142.9% (Table 2), most of the contaminating pesticides remained in the distillers’ stillage. In terms of mass balance, the DMR + DSRR of fenthion, terbufos (0.04 ppm after spiking), and ethoprophos were more than 79.5%. In particular, that of fenthion was about 87% whether the amount of contamination was 0.14 or 0.33 ppm. In addition, iprobenfos and flutolanil showed DMR + DSRR in a range of 139–142.9% and terbufos (0.02 ppm after spiking) showed somewhat low DMR + DSRR (i.e., about 50%).

Previous studies indicated that the migration degrees of pesticides into distillates or distilled spirits differ [3,4,5]. Inoue et al. [5] showed that higher vapor pressures of the pesticide component resulted in higher migration into distillates. In addition, they suggested that the molecular weight and chemical stability at high temperatures needed to be considered. In Table 2, the vapor pressure (46.5 mPa at 26 °C) of ethoprophos, which exhibited the highest DMR, was higher than that of the other pesticides while the molecular weight (242.3 g/mol) was the lowest. In contrast, the DMR of flutolanil with the largest contamination (1.27 ppm) was very low (0.8%). These results can be attributed to the fact that flutolanil has a low vapor pressure (1.77 mPa at 25 °C) and the highest molecular weight (323.3 g/mol) of all pesticides tested. However, this trend did not always hold true. For instance, terbufos with a molecular weight of 288.4 g/mol and vapor pressure of 34.6 mPa (at 25 °C) was not detected or only trace amounts were detected, although its relatively low contamination amounts (0.02 and 0.04 ppm) may be claimed. Therefore, along with vapor pressures and molecular weights, the physicochemical properties of the pesticide (e.g., solubility in water or ethanol; binding with or adsorption to certain components in the distillers’ stillage, etc.) likely affected the migration.

### 2.3. Determination of Six Pesticides from Pilot-Plant Scale of Distillation Column

**Figure 4:**
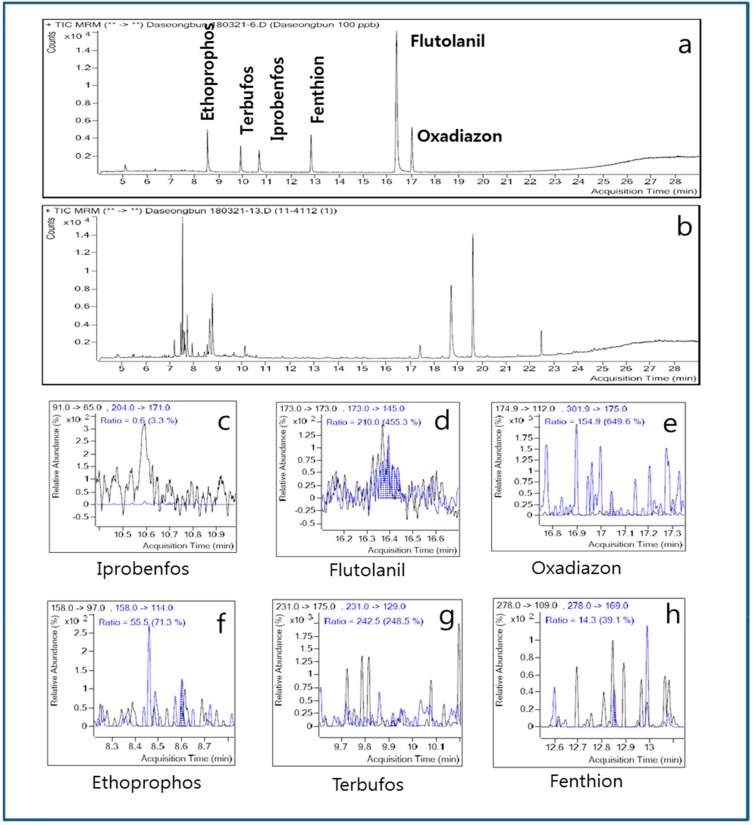
Identification of contaminating pesticides in the distilled spirits obtained from the pilot-plant scale distillation column. (**a**) Standards; (**b**) distilled spirits; (**c**) iprobenfos; (**d**) flutolanil; (**e**) oxadiazon; (**f**) ethoprophos; (**g**) terbufos; (**h**) fenthion.

The pilot-plant scale distillation column (similar to the Patent or Coffey still method) was used to determine whether the contaminating pesticides migrate to the distilled spirits (Figure 1b,c). The distillation column is mainly used by manufacturers of distilled spirits and six pesticides were selected to contaminate the fermented liquefaction, as shown in Table 3. None of the six pesticides (i.e., fenthion, terbufos, ethoprophos, iprobenfos, oxadiazon, and flutolanil) used for contamination of the fermented liquefaction were detected in the distilled spirits. From Figure 4, no pesticide showed an ion pattern with a matching rate sufficient for identification, suggesting that none of the six pesticides could be detected in the distilled spirits composed of 95.7 vol% ethyl alcohol.

In the distillers’ stillage, fenthion, terbufos, and ethoprophos were not detected, whereas iprobenfos, oxadiazon, and flutolanil were detected (Table 3). In particular, iprobenfos that was spiked into the fermented liquefaction to a final content of 0.8 ppm was found at 0.43 ppm in the distillers’ stillage. In addition, flutolanil and oxadiazon, which were spiked to a final content of 4 and 0.2 ppm, were detected at only 1.4 and 0.01 ppm in the distillers’ stillage, respectively (Table 3). Therefore, the results in Table 3 could not fully explain the fact that the pesticides were not detected in the distilled spirits because most of them remained in the distillers’ stillage. A possible reason is that sample homogeneity could be hardly secured from the distillers’ stillage where pesticides can bind with or adsorb to certain components in the distillers’ stillage. However, since distilled spirits mostly consist of ethanol, its constituents are not as complex as distillers’ stillage, and thus homogeneity can be achieved. Although the distillation temperatures can be also considered to be the reason, the pesticides used in this study are generally stable at temperatures of around 100 °C [10]. In conclusion, unlike cases where some pesticides migrate into the distillate during single-stage distillation, none of the six pesticides were detected in the distilled spirits obtained though the pilot-plant scale distillation column.

Water and ethanol coexist in the fermented liquefaction and are not be completely separated by distillation. Theoretically, distilled spirits are azeotropic mixtures of 95.6% ethanol and 4.4% water [12]. Although distillates containing more water than ethanol should have been obtained via single-stage distillation, the obtained distilled spirits were mostly composed of ethanol. Cabras et al. [4] suggested that the migration of large amounts of contaminants would occur in water vapor rather than ethanol vapor and, thus, the distilled spirits produced commercially would be free from the residual pesticides used in their study. Meanwhile, ethanol and water act as carriers for all volatile substances during distillation. Initially, a large amount of ethanol emerges along with highly volatile substances. However, the amount of ethanol gradually decreases with increasing water over time, and low volatility substances may emerge with water [12]. Therefore, under single-stage distillation conditions (i.e., a large amount of water distillated together with a small amount of ethanol), it is possible that some pesticides can migrate into the distillate with water vapor, depending on their physicochemical properties. In contrast, migration of the pesticides to the distilled spirits obtained from the distillation column should be extremely limited because the water vapor was condensed and flowed down in the five sectors irregularly filled with copper chips while the ethanol vapor flowed upwards, resulting in the effective separation of ethanol from water (Figure 1b).

As the results show, during single-stage distillation, some pesticides transferred to the distillate due to their physiochemical characteristics. Therefore, the distillation column was employed to obtain the distilled spirits, which is the preferred distillation method in the manufacturing industry. We contaminated the fermented liquefaction with pesticides at high concentrations and obtained the final product of distilled spirits. The results revealed that the contaminated pesticides did not remain in the distilled spirits. It is presumed that concentrating ethanol to 95% or more during the distillation process excludes the possibility of pesticide residues in distilled spirits.

## 3. Materials and Methods

### 3.1. Materials

The rice bran powder, coenzyme, liquefying enzyme solution, and diastatic enzyme solution were provided by the Korea Alcohol and Liquor Industry Association (KALIA). The Pb and Cd standard solution (1000 ppm in 0.5 N HNO_3_ solution, respectively) were purchased from Kanto Chemical Company (Tokyo, Japan). Commercial agricultural pesticides containing terbufos (labeled as 3%) and fenthion (labeled as 50%), and iprobenfos (labeled as 48%) and flutolanil (labeled as 15%) were purchased from Farmhannong Inc. (Seoul, Korea) and Kyung Nong Inc. (Seoul, Korea), respectively. Also, commercial pesticide products containing ethoprophos (labeled as 5%) and oxadiazon (labeled as 12%) were purchased from Dongbang Agro Inc. (Seoul, Korea).

### 3.2. General Experimental Procedures

Two types of distillation process were performed: single-stage distillation and pilot-plant scale distillation. For single-stage distillation, a small-scale distillation apparatus was used to determine the mass balance, in which five pesticides were artificially added to the raw material (i.e., rice bran powder). To obtain the mass balance, an experimental scheme (Figure 1a) was first developed where Pb and Cd (i.e., materials that are not transferred into distillate during distillation) were used to verify the distillation mass balance. Detailed information regarding these procedures is provided in the Materials and Methods Section 3.3, Section 3.4, Section 3.5, Section 3.6 and Section 3.7. For the pilot-plant scale distillation column, six pesticides were artificially added to the fermented liquefaction and the detailed procedures are described in the Materials and Methods Section 3.8. The contents of the obtained distillate, distillers’ stillage (both from the single-stage distillation) and distilled spirits (from the pilot-plant scale distillation column) were analyzed via GC-MS/MS according to Section 3.9.

### 3.3. Sample Preparation of Pb and Cd Contamination for Ethanol Fermentation

After adding 10 g of rice bran powder into a 500-mL round flask, each of Pb and Cd standard solutions (1000 ppm) were added to reach 10 ppm, respectively. By adding 22 mL of distilled water, 0.37 mg of ammonium sulfate and 4.625 μL of liquefaction enzyme solution, the steaming process was carried out by heating for 20 min at 85 °C followed by heating for 1 h at 95 °C. Then, the temperature of the mixture was lowered to 70 °C and maintained for 30 min, thereafter 37 μL of the diastatic enzyme solution, 3.7 mg of the coenzyme and 2 mL of distilled water was added and the mixture was heated at 70 °C for 1 h. After cooling to 33 °C, 3 mL of distilled water and 3.7 mL of the prepared *Saccharomyces cerevisiae* (Jenico Instant yeast 1 g/5 mL, cultured at 37 °C for 20 min) were added, and the fermentation process was carried out at 32 °C for 4 d. During the fermentation process, the round flask was closed with a coak.

### 3.4. Pb and Cd: Preparation of Distillate and Distillers’ Stillage from Single-Stage Distillation

When the fermentation was completed, the 500 mL round flask was fitted on a rotary vacuum evaporator (EYELA, Rotary Evaporator N-1000, Digital Water bath SB-1000, Aspirator A-3S, Tokyo, Rikakikai Co. Ltd., Japan) to carry out single-stage distillation to obtain the distillate and distillers’ stillage (Figure 1a). The single-stage distillation was carried out while maintaining a temperature of 80 °C, pressures of 50–70 cmHg, and a condenser temperature of –10 °C. After obtaining about 20 mL of distillate through the distillation of the 500 mL round flask from fermentation under the abovementioned conditions, 20 mL of additional distilled water was added to the round flask and distillation was continued to obtain about 10 mL of additional distillate. The foregoing process was repeated twice to obtain about 40 g of distillate. Before distilling other samples, 200 mL of distilled spirits (95% ethanol) was distilled to wash the inside of the evaporator. The distillate obtained by performing single-stage distillation was transferred to a wide-mouthed beaker and left in an oven maintained at 54 °C until the weight was reduced to about 10 g. Meanwhile, the distillers’ stillage remaining in the flask was collected from the flask as much as possible while washing the flask several times using water and sonication. The collected distillers’ stillage was transferred to a beaker and dried in an oven. By measuring the weight periodically, it was dried until the weight was reduced to about 10 g. Therefore, the rice bran powder as raw material (10 g), the distillate (10 g), and the distillers’ stillage (10 g) were analyzed for measuring the contents of Pb and Cd, respectively. The contents (ppm) were expressed as the distillate migration rate (DMR) and the distillers’ stillage residual rate (DSRR) as follows: DMR (%) = (content detected from the distillate / content detected from the rice bran powder after spiking) × 100; DSRR (%) = (content detected from the distillers’ stillage / content detected from the rice bran powder after spiking) × 100.

### 3.5. Analysis of Pb and Cd by Inductively Coupled Plasma (ICP)

Analysis of Pb and Cd was carried out according to the Korean Food Standards Codex [13]. Briefly, the samples were soaked in decomposition beaker containing 20 mL of HNO_3_ for 24 h. Thereafter, each beaker was heated up to 180 °C for 2 h. The completely decomposed samples were cooled at room temperature, and the filtrate was obtained using Watman no. 2 filter paper. Then, the filtrate was massed up to 100 mL with deionized water for quantification.

The Pb and Cd were quantified using ICP-OES (iCAP 7000 series, Thermo Scientific, Cambridge, UK). The analysis conditions of ICP-OES were as follows: RF power, 1,350 w; nebulizer gas flow, 0.5 L/min; auxiliary gas flow, 0.5 L/min; sample pump speed, 50 rpm; measure mode, axial. Pb and Cd were measured at a 220.353 nm and 226.502 nm wavelength spectrum, respectively. The contents were quantified with the calibration curves obtained by six concentrations (0.1, 0.5, 1, 2, 5, 10 ppm). The calibration curve of Pb was Y= 0.9992X (R^2^ = 1), and that of Cd was Y= 1.004X (R^2^ = 0.9999).

### 3.6. Sample Preparation of Pesticides Contamination for Ethanol Fermentation

It is difficult to accept the pesticide content labeled on commercial products as the exact amount contained in the product. Therefore, the experiment was carried out as follows. Pesticides solutions were prepared by diluting with distilled spirits (alcohol 95%) to a concentration of 100 μg/mL from the contents labeled on the products. Then, each prepared pesticides solution was spiked into rice bran powder at the same time, and the actual contamination amount was measured. A more detailed description is as follows. First, 5 g of rice bran powder was put into a 500 mL round flask, weighed, and spiked with terbufos, iprobenfos, fenthion, and flutolanil. The amount contaminated with rice bran powder after spiking was 5.1–11.2 times the maximum residue limits (MRL) [10]. The steaming process was initiated by adding 13.5 mL of distilled water, 0.185 mg of ammonium sulfate, and 2.315 μL of the liquefying enzyme solution at 85 °C for 20 min followed by heating at 95 °C for 1 h. Thereafter, the temperature was lowered to 70 °C. To this end, 18.5 μL of diastatic enzyme solution and 1.85 mg of the coenzyme were added to the mixture and heated for 1 h at 70 °C. Thereafter, the mixture was cooled to 33 °C and 1.85 mL of the prepared *Saccharomyces cerevisiae* (Jenico Instant yeast 1 g/5 mL, activated for 20 min at 37 °C) were added to the mixture. The fermentation process was carried out for 4 d at 32 °C. During the fermentation, the round flask was closed with a coak. In addition, to contaminate a greater quantity, rice bran powder was simultaneously spiked with ethoprophos, terbufos, and fenthion. Then, fermentation was carried out in the same method as mentioned above. The amount contaminated with rice bran powder after spiking was 16–70 times the MRL [10].

### 3.7. Pesticides: Preparation of Distillate and Distillers’ Stillage from Single-Stage Distillation

After completion of the fermentation, single-stage distillation was carried out with the 500-mL round flask using a rotary vacuum evaporator (EYELA, Rotary Evaporator N-1000, Digital Water bath SB-1000, Aspirator A-3S, Tokyo, Rikakikai Co. Ltd., Japan) under the conditions previously described to obtain the distillate and distiller stillage. First, the fermented liquefaction contaminated with terbufos (0.02 ppm after spiking), iprobenfos, fenthion (0.14 ppm after spiking), and flutolanil was distilled. After obtaining the distillate, a total of 35 mL of new distilled water was subsequently added to continue distillation. During the distillation, some of the distillate remained in the condenser and trap of a rotary vacuum evaporator, especially at the first time of distillation. Thus, 12 g of the distillate along with 19.7 g of distillers’ stillage were obtained. Because most of the water instead of ethanol was distilled during the distillation, the obtained distillate was added with water to become 20 g due to the mass balance. Before distilling the next sample, 200 mL of distilled spirits (95% ethanol) was used to wash the evaporator. Then, the sample contaminated with ethoprophos, terbufos (0.04 ppm after spiking) and fenthion (0.3 ppm after spiking) was distilled. After obtaining about 10 mL of the distillate, 15 mL of new distilled water was added to the round flask and distillation was continued to obtain an additional distillate. Thereafter, 15 mL of new distilled water was added to the round flask and distilled continuously. When distillation was finished, about 16 g of distillate along with 20.7 g of distillers’ stillage were obtained. As mentioned above, water was added to the distillate to be 20 g. Then, the distillate was immediately transferred to a 50-mL vial, sealed, and sent to a Korea Advanced Food Research Institute (KAFRI). KAFRI is a specialized analysis institute certified by the Ministry of Food and Drug Safety of Korea. Each flask remained with distillers’ stillage was also sent to a KAFRI as it was, and the entire quantity was used for analysis. Meanwhile, the rice bran powder (5 g) spiked with pesticides was added with distilled water so that the total weight became 20 g before being sent to a KAFRI. In this way, mass balance can be adjusted and the effects of water on the matrix could be minimized. Therefore, the weight of contaminated rice bran powder with water, the distillate, and the distillers’ stillage was about 20 g. After obtaining the contents (ppm) of pesticides in the samples, DMR and DSRR were calculated as follows: DMR (%) = (content detected from the distillate / content detected from the rice bran powder after spiking) × 100; DSRR (%) = (content detected from the distillers’ stillage / content detected from the rice bran powder after spiking) × 100.

### 3.8. Preparation of Distilled Spirits from Pilot-Plant Scale Distillation Column

The distillation column (Figure 1b,c), which is mainly used by distilled spirits manufacturing companies, was used for distillation on a pilot-plant scale. Using the distillation column, the fermented liquefaction simultaneously contaminated with six pesticides (fenthion, terbufos, ethoprophos, iprobenfos, oxadiazon, and flutolanil) was distilled. Then, the contaminated fermented liquefaction, distillate, and distillers’ stillage were analyzed to examine the content of the pesticides. Each pesticide at a calculated amount of 16 times the MRL [10] was spiked into 5 kg of the fermented liquefaction (11.3% ethanol). Such contamination amount was calculated according to the contents labeled on the commercial products. When necessary, pesticides solutions diluted with distilled spirits were prepared before spiking. Thereafter, 1.3 kg of the contaminated fermented liquefaction was placed in a distillation vessel that was maintained at 104.5–105.5 °C. The temperature at the top of the distillation column and condenser was maintained at 77.8–78.0 °C and at 50 °C, respectively. The distillation column was made of stainless steel, having 25 mm in diameter and 2000 mm in total height. It consisted of five sections and each section was irregularly filled with copper chips (0.25 inch). The capacity was designed to produce 4.1 L of distilled spirits (ethanol content 95%) by feeding 36 L of fermented liquefaction (ethanol content 11%). After distillation, 20 g was taken from 90 mL of the distilled spirits (95.7% ethanol content), and 20 g of distillers’ stillage was also taken to be analyzed by KAFRI.

### 3.9. Analysis of Pesticides by GC-MS/MS

The contaminated rice bran powder after spiking, distillates, distilled spirits and distillers’ stillage were analyzed according to Korean Food Standards Codex [14]. In short, the sample mixed with acetone was extracted and the layer separated using organic solvents (e.g., petroleum ether, dichloromethane, etc.) were dehydrated with an anhydrous sodium sulfate column. After concentration using a Kuderna-Danish apparatus, a florisil cartridge (Agela Tech, Torrance, CA, USA) was applied. In another way, the sample mixed with acetonitrile was extracted and dehydrated with an anhydrous sodium sulfate column. After vacuum evaporation, the sample was re-dissolved with a mixed organic solvent (i.e., acetone and n-hexane). Thereafter, the sample was purified using a florisil cartridge, filtered with membrane filters (PTFE 0.45 μm), and analyzed with GC-MS/MS.

The samples from the single-stage distillation experiment were analyzed under the following conditions. Pesticide analysis was performed using a gas chromatograph (Agilent 7890A, Santa Clara, CA, USA) equipped with a triple quadrupole mass spectrometer (Agilent 7000, Santa Clara, CA, USA). The column used was HP-5MS (30 m × 0.25 mm × 0.25 μm, Agilent, Torrance, CA, USA) and the operating conditions were as follows: initial temperature, 100 °C (1 min), increased by 30 °C/min to 300 °C for 3 min; inlet temperature, 275 °C; He carrier gas (splitless, flow rate: 1 mL/min); source temperature, 300 °C; quadrupole temperature, Q1 and Q2 150 °C; collision gas flow, nitrogen at 1.5 mL/min, and helium, at 2.25 mL/min. Mass spectra were recorded in the MRM mode. The precursor ion, product ion, and collision energy for identification of each pesticide are as follows: Ethoprophos = 158 (precursor ion, *m/z*), 114 (product ion, *m/z*), 2 (collision energy, eV); fenthion = 278, 169, 14; flutolanil = 173, 145, 10; iprobenfos = 204, 171, 2; terbufos = 231, 129, 20; oxadiazon = 301.9, 175, 13. In addition, the precursor ion, product ion and collision energy for quantification of each pesticide ingredient are as follows. Ethoprophos = 158 (precursor ion, *m/z*), 97 (product ion, *m/z*), 10 (collision energy, eV); fenthion = 278, 109, 14; flutolanil = 173, 173, 2; iprobenfos = 91, 65, 14; terbufos = 231, 175, 2; oxadiazon = 174.9, 112, 15. In addition, the samples from the distillation column experiment were analyzed under the following conditions. The oven temperature was held for 2 min at 60 °C, ramped to 165 °C at 30 °C/min, increased to 195 °C at 15 °C/min, and then held for 1 min, ramped to 210 °C at 2 °C/min, increased to 220 °C at 5 °C/min, and finally increased to 300 °C (hold 3 min) at 10 °C/min with a flow of 1.0 mL/min.

## 4. Conclusions

In this study, we examined the fate of pesticide ingredients during distillation, which is one of the important processes in manufacturing distilled spirits. Pesticide ingredients were added at high concentrations into the raw material (i.e., rice powder) and the fermented liquefaction, and two different distillations were performed to identify whether any of the pesticide ingredients remain and the residue level at which they were present in the distillate and distilled spirits. In particular, we examined the mass balance of the raw materials, distillate and distillers’ stillage, during the process. Based on the law of conservation of mass, the mass balance reveals whether the levels of pesticides in the contaminated raw material and the distillate are comparable. In addition, the mass balance could be used to verify and correct qualitative and quantitative analysis errors on trace components. For this purpose, an experimental scheme was designed, as shown in Figure 1a, using heavy metals (Pb and Cd that are not transferred into distillate during distillation) as potential trace components. Although Pb and Cd do not expect to be transferred to the distillate by distillation like pesticides, they are also hazardous compounds that should be supervised for consumers.

In summary, rice bran powder was spiked with Pb and Cd at 10 ppm thereby the contents were 50 and 100 times the MRL, respectively. Pb and Cd were not detected from the distillate obtained through the single-stage distillation, showing 103–114% of the residual rates in the distillers’ stillage. When five pesticide ingredients (terbufos, fenthion, iprobenfos, flutolanil, and ethoprophos) were contaminated to reach about 5.1–70 times the MRL, DMR was 0–14.3%, suggesting that some pesticides migrated to the distillate during the single-stage distillation. Meanwhile, when a pilot-scale of distillation column was used for distillation, six pesticides (fenthion, terbufos, ethoprophos, iprobenfos, oxadiazon, and flutolanil) were contaminated in fermented liquefaction, in which a contaminating concentration was predicted to be 16 times the MRL. However, none of the pesticides were detected from the distilled spirits, suggesting that the pesticides hardly migrate to the distilled spirits.

Contamination amounts of the pesticides used in this study are not expected to occur in industrial practice. Nevertheless, distillation effectively reduced the residues of the studied pesticides in the distillate and especially in the distilled spirits. Therefore, it was assumed that the risk to the distilled spirit posed by pesticides can be minimized with proper distillation. The results of this study are expected to contribute to the risk management of pesticide contamination in the industry and could improve food safety to meet the continual increase in demand from consumers.

## Figures and Tables

**Table 1:** Content (ppm), distillate migration rate (%, DMR), and distillers’ stillage residual rate (%, DSRR) of the contaminated lead (Pb) and cadmium (Cd) from the single-stage distillation experiment.

	Pb	Cd
Limit of detection (LOD) (ppm)	0.001	0.001
Limit of quantification (LOQ) (ppm)	0.01	0.01
Spiking content (ppm)	10	10
Content in rice bran powder before spiking (ppm)	0.017	0.011
Content in rice bran powder after spiking (ppm)	8.85	9.27
Content in distillate (ppm)	ND ^a^	ND
Distillate migration rate (%) ^b^	0	0
Content in distillers’ stillage (ppm)	10.09	9.57
Distillers’ stillage residual rate (%) ^c^	114	103

^a^ Not detected; ^b^ DMR (%) = (content detected from the distillate/content detected from the rice bran powder after spiking) × 100; ^c^ DSRR (%) = (content detected from the distillers’ stillage/content detected from the rice bran powder after spiking) × 100.

**Table 2:** Content (ppm), distillate migration rate (%, DMR), and distillers’ stillage residual rate (%, DSRR) of contaminated pesticides from the single-stage distillation experiment.

	Vp ^a^ mPa (at Temp.)	Mw ^b^ (g/mol)	Log P (Solubility to Water at Temp.)	LOQ (ppm)	LOD (ppm)	Rice Bran Powder after Spiking	Distillate (ppm)	DMR (%) ^g^	Distillers’ Stillage (ppm)	DSRR (%) ^h^
Terbufos	34.6 mPa (25 °C)	288.4	2.77 (4.5 mg/L at 27 °C)	0.005	0.002	0.02	ND ^d^	0	0.01	50
Iprobenfos	12.2 mPa (25 °C)	288.3	3.37 (0.54 g/L at 20 °C)	0.009	0.003	0.28	ND	0	0.40	100 < (142.9)
Fenthion	1.4 mPa (25 °C)	278.3	4.84 (4.2 mg/L at 20 °C)	0.007	0.002	0.14	Trace ^e^ (0.0024)	1.7	0.12	85.7
Flutolanil	1.77 mPa ^c^ (25 °C)	323.3	3.17 (8.01 mg/L at 20 °C)	0.007	0.002	1.27	0.01	0.8	1.77	100 < (139)
Ethoprophos	46.5 mPa (26 °C)	242.3	3.59 (700 mg/L at 20 °C)	0.002	0.005	0.35	0.05	14.3	0.30	85.7
Terbufos	34.6 mPa (25 °C)	288.4	2.77 (4.5 mg/L at 27 °C)	0.005	0.002	0.04	Trace ^f^ (0.0018)	4.5	0.03	75
Fenthion	1.4 mPa (25 °C)	278.3	4.84 (4.2 mg/L at 20 °C)	0.007	0.002	0.33	0.01	3	0.28	84.8

^a^ Vapor pressure [10]; ^b^ Molecular weight [10]; ^c^ Vapor pressure is obtained after unit conversion from Reference [11], and its vapor pressure at 20 °C is 4.1 × 10^−4^ mPa [10]; ^d^ Not detected; ^e^ The value below LOQ (i.e., trace) was compulsively integrated; ^f^ The value similar to LOD was marked as trace in this time and was compulsively integrated; ^g^ DMR (%) = (content detected from the distillate / content detected from the rice bran powder after spiking) × 100; ^h^ DSRR (%) = (content detected from the distillers’ stillage / content detected from the rice bran powder after spiking) × 100. All pesticides used in this study were not detected in the rice bran powder used as a raw material.

**Table 3:** Content (ppm) of contaminated pesticides in the distilled spirits and distillers’ stillage from the pilot-plant scale in the distillation column experiment.

	Fenthion	Terbufos	Iprobenfos	Flutolanil	Oxadiazon ^b^	Ethoprophos
Spiking content (ppm)	0.2	0.04	0.8	4.0	0.2	0.08
Content in distilled spirits (ppm)	ND ^a^	ND	ND	ND	ND	ND
Content in distillers’ stillage (ppm)	ND	ND	0.43	1.4	0.01	ND
Ethanol volume % in distilled spirits	95.7					

^a^ Not detected; ^b^ LOD and LOQ are 0.002 and 0.007 mg/kg, respectively. Other pesticides are presented in Table 2.
